# Screen time increases overweight and obesity risk among adolescents: a systematic review and dose-response meta-analysis

**DOI:** 10.1186/s12875-022-01761-4

**Published:** 2022-06-28

**Authors:** Purya Haghjoo, Goli Siri, Ensiye Soleimani, Mahdieh Abbasalizad Farhangi, Samira Alesaeidi

**Affiliations:** 1 Urology Research Center, Razi Hospital, School of Medicine, Guilan University of Medical Sciences, Rasht, Iran; 2 Department of Internal Medicine, Amir Alam Hospital, Tehran University of Medical Sciences, Tehran, Iran; 3 Student Research Committee, Tabriz University of Medical Sciences, Tabriz, Iran; 4 Department of Community Nutrition, Faculty of Nutrition, Tabriz University of Medical Sciences, Tabriz, Iran; 5 Rheumatology Research Center, Tehran University of Medical Sciences, Tehran, Iran

**Keywords:** Screen time, Overweight, Obesity, Dose-response, Adiposity, Meta-analysis

## Abstract

**Background:**

Adolescence is a critical period in human life, associated with reduced physical activity and increased sedentary behaviors. In this systematic review and dose-response meta-analysis, we evaluated the association between screen time and risk of overweight/obesity among adolescents.

**Methods:**

A systematic search in electronic databases, including PubMed, Embase, and Scopus was performed up to September 2021. All published studies evaluating the association between screen time and risk of overweight/obesity among adolescents were retrieved. Finally, a total of 44 eligible studies were included in the meta-analysis.

**Results:**

The results of the two-class meta-analysis showed that adolescents at the highest category of screen time were 1.27 times more likely to develop overweight/obesity (OR = 1.273; 95% CI = 1.166–1.390; *P* < 0.001; I-squared (variation in ES attributable to heterogeneity) = 82.1%). The results of subgrouping showed that continent and setting were the possible sources of heterogeneity. Moreover, no evidence of non-linear association between increased screen time and risk of overweight/obesity among adolescents was observed (P-nonlinearity = 0.311).

**Conclusion:**

For the first time, the current systematic review and meta-analysis revealed a positive association between screen time and overweight/obesity among adolescents without any dose-response evidence.

**Trial registration:**

The protocol of the current work has been registered in the PROSPERO system (Registration number: CRD42021233899).

**Supplementary Information:**

The online version contains supplementary material available at 10.1186/s12875-022-01761-4.

## Background

Adolescence is a critical period regarding physical activity-related behaviors since regular physical activity decreases and sedentary behavior increases in this period [1, 2]. Screen-related physical activities like television watching are very common among adolescents particularly in modern societies; it is reported that adolescents spend about 3h per day on screen activities [3]. Screen time constitutes an important part of adolescents’ life, and they are major TV users [4, 5]. In a study, 57% of adolescents reported watching TV every day (average time in a day: 109 minutes) [6]. According to some recent evidence, increased screen-related sedentary behaviors led not only to obesity growth [7], but also to mental problems among adolescents [8–12].

Sedentary behavior guidelines recommend less than 2h per day of recreational screen time for the youth [13]. However, it has been estimated that more than 50% of adolescents exceed this time [14]. In a report from the Health Behavior in School-Age Children (HBSC), which was performed among adolescents aged 11, 13, and 15 years in 41 European and North American countries, 56–65% of the adolescents spent 2h or more per day watching television [15, 16].

Sedentary behaviors are characterized by activities with low energy expenditure (< 1.5 metabolic equivalents) in a sitting position like television watching or other screen behaviors [17]. Such behaviors are an important risk factor for cardio-metabolic disease in adulthood [18–21]. In adolescents, obesity is associated with dyslipidemia, glucose intolerance, and hypertension [22]. In several population-based studies [23–25], high screen time was positively associated with high blood pressure (BP), high low-density lipoprotein (LDL) cholesterol, and triglyceride (TG) (*P* < 0.05).

Numerous studies have reported the association between screen time and adiposity among adolescents; however, the results are inconsistent. Some studies reported increased odds of obesity by increasing screen time [22, 26, 27]. For example, Cheng [26] included 2201 Chinese adolescents and reported increased odds of obesity for those with more than 2h of screen time per day (1.53; 95%CI = 0.95–2.09; *P* < 0.001). In contrary, in another population-based study in the school setting, Lopez-Gonzalez evaluated 1,319,293 adolescents aged 12–14 years old and reported no significant association between obesity and screen time [28]. Several other studies also reported no association between obesity and screen time [28–31]. Meanwhile, in some other studies, only watching television or playing video games for more than 3h per day increased the risk of obesity among adolescents [32–34]. More surprisingly, in a study by De-Lima et al. [35], a non-significant reduced risk of excess weight was observed by increased screen time of more than 4h per day (*P* = 0.87; 95% CI = 0.59–1.30).

As mentioned, there is an inconsistency between the results of different studies regarding the association between screen time and overweight/obesity among adolescents. In today’s digital age, screen time is almost unavoidable and it has drastically increased among children and adolescents, especially during the coronavirus disease 2019 (COVID-19) pandemic. Excessive screen time may have adverse health consequences because it replaces healthy behaviors and habits like physical activity and sleep routine [36, 37]. However, currently, there is no systematic review and meta-analysis evaluating the association between screen time and obesity among adolescents. More importantly, no study in this filed has focused on such dimensions as the type of screen (TV, PC, DVD, video games, etc.), duration of screen use, and several other factors affecting the association between overweight/obesity and screen time.

Therefore, in this systematic review and meta-analysis, we systematically searched and analyzed all the available literature evaluating the association between overweight/obesity and screen time among adolescents. We also classified the results according to numerous factors, including geographical distribution, screen type, setting, obesity status, as well as the quality and sample size of studies to identify the possible determinants of these associations.

## Methods

The results were reported according to PRISMA (Preferred Reporting Items for Systematic Reviews and Meta-Analyses) checklist (Sup. Table 1) [38]. The protocol’s registration code in PROSPERO is CRD42021233899.

### Search strategy, selection of studies, inclusion and exclusion criteria

**Fig. 1:**
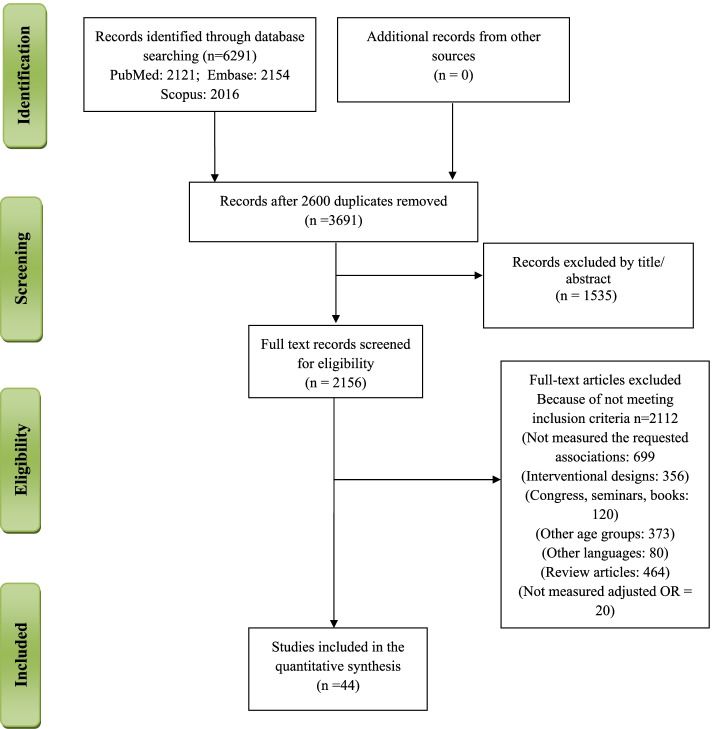
Study Flowchart

A total of 6291 articles were retrieved through searching electronic databases, including PubMed, Embase, and Scopus up to September 2021 (Fig. 1). The search strategy for PubMed is provided in Sup. Table 2, and it has been adopted for each electronic database. A total of 44 manuscripts were eligible to be included in the final meta-synthesis.

The inclusion criteria were as follows: 1) studies with observational designs (case control, cross-sectional or cohort studies with the baseline or cross-sectional measurement of study parameters), 2) studies evaluating the relationship (OR, RR, or HR) between screen time and risk of overweight/obesity, and (3) studies conducted only among adolescents (age ≥ 10–20 years). The studeis that did not provide an OR, RR, or HR or those with adjustment for confounders were excluded from the analysis.

The PICO model (patients, intervention, comparison, outcome), which is one of the most widely used models for formulating clinical questions, was used for selecting the studies (Table 1).

**Table 1:** The PICO criteria used for the systematic review

PICO criteria	Description
Participants	Adolescents population
Exposure (Interventions)	Highest category of screen time
Comparisons	Lowest category of screen time
Outcome	Overweight/ obesity
Study design	Observational studies with the design of cross-sectional, case control or cohort

#### Data extraction and quality assessment

Data extraction was done by two authors in a standard EXCELL datasheet. The data sheet included the following information: name of first author and journal, publication year, country, setting, age range, number of participants, study design, adjusted covariate, gender, definition of overweight/obesity and screen time, overweight/obesity status, weight, height, screen time measurement tools, and main results. Any disagreements between reviewers were resolved by discussion. The methodological quality of studies were assessed using the Agency for Healthcare Research and Quality (AHRQ) checklist [54] (Table 2).

**Table 2:** Agency for Healthcare Research and Quality (AHRQ) checklist to assess quality of the cross-sectional studies

**ARHQ Methodology Checklist items for Cross-Sectional study**	**Zhang Y** **[**39**]**	**De-Lima** **TR [**35**]**	**Zulfiqar T** **[**33**]**	**Kerkadi A** **[**30**]**	**Hu J [99]**	**De-Jong E** **[**40**]**	**Franceschin MJ** **[**22**]**		**Dalamaria, T** **[**27**]**	**Cheng L** **[100]**	
1) Define the source of information (survey, record review)	⊕	⊕	⊕	⊕	⊕	⊕	⊕		⊕	⊕	
2) List inclusion and exclusion criteria for exposed and unexposed subjects (cases and controls) or refer to previous publications	⊕	⊕	⊕	⊕	⊕	⊕	⊕		⊕	⊕	
3) Indicate time period used for identifying patients	⊕	⊕	⊕	_	⊕	⊕	⊕		⊕	⊕	
4) Indicate whether or not subjects were consecutive if not population-based	⊕	–	–	⊕	–	–	–		–	–	
5) Indicate if evaluators of subjective components of study were masked to other aspects of the status of the participants	–	–	–	U	–	–	–		–	–	
6) Describe any assessments undertaken for quality assurance purposes (e.g., test/retest of primary outcome measurements)	–	–	U	U	U	U	U		U	⊕	
7) Explain any patient exclusions from analysis	⊕	⊕	⊕	_	⊕	–	⊕		–	⊕	
8) Describe how confounding was assessed and/or controlled.	⊕	⊕	⊕	⊕	⊕	⊕	–		⊕	⊕	
9) If applicable, explain how missing data were handled in the analysis	⊕	–	⊕	⊕	⊕	–	⊕		⊕	⊕	
10) Summarize patient response rates and completeness of data collection	⊕	⊕	⊕	⊕	⊕	–	⊕		⊕	⊕	
11) Clarify what follow-up, if any, was expected and the percentage of patients for which incomplete data or follow-up was obtained	–	–	–	⊕	–	–	–		–	–	
**Total score**	8	6	7	7	7	4	6		6	8	
**ARHQ Methodology Checklist items for Cross-Sectional study**	**Lopez-GonzalezD [**28**]**	**Pabón D [**41**]**	**Haidar A [**29**]**	**Saha M [**31**]**	**Mansoori M [**42**]**	**Godakanda I [**43]	**Talat MA [**44**]**		**Piryani MA [**44**]**	**Moradi G [**45**]**	
1) Define the source of information (survey, record review)	⊕	⊕	⊕	⊕	⊕	⊕	⊕		⊕	⊕	
2) List inclusion and exclusion criteria for exposed and unexposed subjects (cases and controls) or refer to previous publications	⊕	⊕	⊕	⊕	⊕	⊕	⊕		⊕	⊕	
3) Indicate time period used for identifying patients	⊕	⊕	⊕	⊕	⊕	⊕	⊕		⊕	⊕	
4) Indicate whether or not subjects were consecutive if not population-based	⊕	–	–	–	–	–	⊕		–	–	
5) Indicate if evaluators of subjective components of study were masked to other aspects of the status of the participants	U	U	U	–	–	U	U		U	–	
6) Describe any assessments undertaken for quality assurance purposes (e.g., test/retest of primary outcome measurements)	U	U	U	–	⊕	U	U		U	–	
7) Explain any patient exclusions from analysis	_	_	_	–	⊕	⊕	_		_	–	
8) Describe how confounding was assessed and/or controlled.	⊕	⊕	–	⊕	⊕	⊕	⊕		⊕	⊕	
9) If applicable, explain how missing data were handled in the analysis	⊕	⊕	⊕	–	–	⊕	**–**		–	–	
10) Summarize patient response rates and completeness of data collection	⊕	–	–	–	⊕	–	–		–	–	
11) Clarify what follow-up, if any, was expected and the percentage of patients for which incomplete data or follow-up was obtained	⊕	–	–	–	–	⊕	–		–	–	
Total score	8	5	4	4	7	7	5		4	4	
**ARHQ Methodology Checklist items for Cross-Sectional study**	**Watharkar A [**46**]**	**De- Lucena JMS [**47**]**	**Velásquez-Rodríguez CM [**48**]**	**De Jong E [**40**]**	**Ercan S [**49**]**	**Collins AE [**34**]**	**Drake KM [**50**]**	**Sun Y [**32**]**	**Adesina AF [**51**]**	**El-Gilany AH [**52**]**	**Byun W [**53**]**
1) Define the source of information (survey, record review)	⊕	⊕	⊕	⊕	⊕	⊕	⊕	⊕	⊕	⊕	⊕
2) List inclusion and exclusion criteria for exposed and unexposed subjects (cases and controls) or refer to previous publications	⊕	⊕	⊕	⊕	⊕	⊕	⊕	⊕	⊕	⊕	⊕
3) Indicate time period used for identifying patients	⊕	⊕	⊕	⊕	⊕	⊕	⊕	⊕	⊕	⊕	⊕
4) Indicate whether or not subjects were consecutive if not population-based	–	⊕	–	⊕	–	–	⊕	–	–	⊕	⊕
5) Indicate if evaluators of subjective components of study were masked to other aspects of the status of the participants	–	–	–	–	–	–	–	–	–	–	–
6) Describe any assessments undertaken for quality assurance purposes (e.g., test/retest of primary outcome measurements)	**–**	**U**	–	**U**	**–**	**–**	–	**U**	**–**	⊕	⊕
7) Explain any patient exclusions from analysis	**–**	⊕	⊕	⊕	**–**	**–**	⊕	⊕	⊕	⊕	⊕
8) Describe how confounding was assessed and/or controlled.	**U**	⊕	–	⊕	**U**	**U**	⊕	⊕	**U**	⊕	⊕
9) If applicable, explain how missing data were handled in the analysis	**–**	**–**	⊕	**–**	**–**	**–**	⊕	**–**	**–**	⊕	⊕
10) Summarize patient response rates and completeness of data collection	–	⊕	–	⊕	–	–	⊕	⊕	–	⊕	⊕
11) Clarify what follow-up, if any, was expected and the percentage of patients for which incomplete data or follow-up was obtained	–	–	–	–	–	–	⊕	–	–	–	–
**Total score**	3	7	5	7	3	3	8	6	4	8	8

### Definitions

The Oxford English Dictionary defines screen time as “the time spent using a device such as a computer, or games” [55]. In the current meta-analysis, according to the World Health Organization (WHO), screen time was defined as “the time spent passively watching screen-based entertainment (TV, computer, and mobile devices); this does not include active screen-based games where physical activity or movement is required” [56]. Therefore, TV watching, smart phone use, internet and computer use, and video games that are played in sedentary position are considered as screen time. As previously described by the WHO, adolescence is defined as the age range of 10–19 years old [57]. Overweight and obesity were defined as follows: (a) as Z score for the body mass index (BMI) for age with the cut-off points of > 1 to ≤2 standard deviations for overweight and values > 2 standard deviations for obesity [58]; (b) as the international age and sex specific cut-offs of BMI [≥85th percentile and less than 95th percentile for overweight and ≥ 95th for obesity [46]; and (c) as BMI cut-off of overweight 25 ≤ BMI ≤ 30 kg/m^2^ and obesity BMI ≥ 30 kg/m^2^ [59].

### Statistical analysis

STATA version 13 (STATA Corp, College Station, TX, USA) was used for data analysis and *P*-values less than 0.05 were considered as statistically significant. The studies reporting the odds ratio (OR) of overweight/obesity in people with highest versus lowest screen time were included in the two-class dose-response meta-analysis. In the two-class meta-analysis, the pooled OR with 95% confidence intervals (CI) were estimated using a weighted random-effect model (the DerSimonian-Laird approach). If the number of participants in the categories were not provided, equal number of participants in each category was assumed. Cochran’s Q and I-squared tests were used to identify between-study heterogeneity as follows: I^2^ <25%, no heterogeneity; I^2^ = 25–50%, moderate heterogeneity; and I^2^ > 50%, high heterogeneity [60].

The possible sources of heterogeneity were identified using subgrouping approach. For subgrouping, the possible confounders were chosen (e.g., continent, screen type, age group, setting, overweight/obesity status, sample size, and quality of study). Begg’s funnel plot was used to evaluate the publication bias followed by the Egger’s regression asymmetry test and Begg’s adjusted rank correlation for formal statistical assessment of funnel plot asymmetry. Because of an evidence of publication bias, trim-and-fill method was used for estimating potentially missing studies due to publication bias in the funnel plot and adjusting the overall effect estimate. For dose-response meta-analysis, only the studies that reported at least three categories for screen time and the odds of overweight/obesity were included in the dose-response meta-analysis. Accordingly, 13 different studies published in five articles were included [61–73]. The median point in each screen time category was identified; when medians were not reported, approximate medians were estimated using the midpoint of the lower and upper limits. When the lowest or highest screen time categories were open-ended, the screen time was calculated by assuming the similar interval for those categories and estimating the mid-point. The reference category was the lowest one, assuming OR and CIs of 1 for it. The potential non-linear associations were assessed using random-effects dose-response meta-analysis by defining the restricted cubic splines with three knots at fixed percentiles (10, 50, and 90%) of distribution, and were used to calculate study-specific ORs.

## Results

### Study characteristics

General characteristics of included studies are represented in Table 3. In the meta-analysis of the odds of overweight/obesity among high screen-user adolescents, a total of 44 studies were included. Also, some of the studies reported the results separately for both genders [28], or reported the separate results for each of the screen types [31, 33, 43, 46, 74], or according to overweight/obesity status [29, 41]. The study by Velásquez-Rodríguez [48] reported separate results for healthy adolescents and adolescents with insulin resistance. Generally, the studies had a cross-sectional design, or cross-sectional data from cohort studies were used for data analysis [32]. The age range of the participants in the included studies was 10–19 years old. The studies had been performed in the United States [29, 41, 43, 74], Brazil [22, 27, 35, 47], Egypt [44, 52], China [26, 39], Iran [45], Indonesia [34], Japan [32], Nigeria [51], Pakistan [42], Nepal [75], Bangladesh [31], Qatar [30], Australia [33], Mexico [28], India [46], Finland [48], Netherland [40], Turkey [49], England [50], and South Korea [53]. The screen time was assessed by validated questionnaires and overweight and obesity definitions were according to (a) as Z score for the BMI for age with the cut-off points of > 1 to ≤2 standard deviations for overweight and values > 2 standard deviations for obesity [58]; (b) as the international age and sex specific cut- offs of BMI [≥ 85th percentile and less than 95th percentile for overweight and ≥ 95th for obesity [46] and (c) as BMI cut-off of overweight 25 ≤ BMI ≤ 30 kg/m^2^ and obesity BMI ≥ 30 kg/m^2^ [59]. All the studies included in the meta-analysis reported an adjusted OR that was adjusted according to the confounders, including age, gender, race, nationality, dietary behaviors, parents’ education, occupation, and socio-economic status.

**Table 3:** The characteristics of studies that evaluated the association between overweight and obesity risk by increased screen time among adolescents

Journal/ Year/ First author	Country	Setting/ num	Design	Age (y)/ gender	Overweight/ obesity status and definition	ST definition	Adjusted variables	Main findings
Revista Paulista de Pediatria/ 2021/ Dalamaria T [27]	Brazil	School/ 1387	Cross-sectional	14–18/ both	Obesity/ ≥85th percentile of age	Internet addiction	None	Increased odds of obesity in internet addicted adolescents [OR = 1.1; CI = 0.9–3.18]. Not adjusted
BMC Public Health/ 2020/ Zhang Y [39]	China	School/ 2264	Cross-sectional	12–15/ both	Obesity/ ≥85th percentile of age	TV, VG, PC	Age, sex, being the single child, ethnic minority, fruit and vegetable intake, sleep time, parents’ Education, fathers’ occupation.	Non-significant association between screen time and odds of obesity.
Nutrients/ 2020/ Lopez-Gonzalez D [28]	Mexico	School/309 girl; 340 boys	Cross-sectional	12–17/ both	Overweight/ obesity defined as ≤95th and ≥ 85th and ≥ 95th percentile of age respectively	TV, electronic games	Stratified by age and sex	Non-significant association between obesity and screen time.
Rev Bras *Cineantropometri Desempenho* Hum/ 2020/ Franceschin MJ [22]	Brazil	School/ 1015	Cross-sectional	15.3/ both	Overweight/ obesity defined as 1 ≤ BMI Z-score < 2	TV, Video game or PC	Sex, age, type of school attended and dietary energy intake	A significant increased odds of overweight/ obesity in those with more than 2 hours per day TV watching (1.73 (1.24–2.42). The OR for PC and video games was 1.01 (0.71–1.45).
Revista Paulista de Pediatria/ 2020/ De Lima TR [35]	Brazil	School/ 583	Cross-sectional	11–17/ both	Overweight defined as BMI Z-score ≥ 1	TV, Video game or PC	Gender, maternal schooling, alcohol consumption, smoking, screen time-sedentary behavior	Non-significant reduced risk of excess weight by increased screen time of more than 4 hours/day (0.87 CI = 0.59–1.30)
Public Health Nutrition/ 2020/ Cheng L [26]	China	School/ 2201	Cross-sectional	10/ both	Obesity/ ≥95th percentile of age	TV/video games/ PC/iPad/ phone	Sex, age and school location (rural or urban) with school as a random effect	Increased odds of obesity for those with more than 2 hours/ d screen time (1.53; CI = 0.95–2.09)
J Immigrant Minor health/ 2019/ Zulfiqar T [33]	Australia	Community/ 2115 girls and 2000 boys	Cross-sectional	10–11/ both	Overweight/ obesity +BMI ≥ 25 kg/m^2^	TV, electronic games	Sleep issues, breastfeeding, birth weight, siblings, foreign language spoken at home, maternal work status, family SEP, maternal partnership status	TV watching of more than 3 hours/ day in weekends was associated with odds of obesity in boys (1.4 (1.0,1.9) and girls (1.5 (1.1,1.9) *P* < 0.05
In J Environ Res Pub Health/ 2019/ Kerkadi A [30]	Qatar	Community/ 1161	Cross-sectional	14–18/ both	Overweight 25 ≤ BMI ≤ 30 kg/m^2^ and obesity BMI ≥ 30 kg/m^2^	TV, Video game or PC	Age, nationality	No significant association between screen time of more than 2 hours/ day and risk of overweight/ obesity (OR = 1; CI = 0.7–1.4)
Plos One/ 2019/ Pabon et al. [41]	USA	Community/ 2358 + 546	Cross-sectional	13–17/ both	Overweight/ obesity defined as 1 ≤ BMI Z-score < 2	TV, Video game	Age, sex, socioeconomic level, geographic area, ethnic group and exposure to television and / or video games.	No significant association between increased screen time and risk of overweight or obesity.
BMC Public Health/ 2019/ Haidar A [29]	USA	School/ 6716	Cross-sectional	14.88/ both	Overweight/ obesity defined as ≤95th and ≥ 85th and ≥ 95th percentile of age respectively	TV, DVD, movies	Grade, gender, ethnicity, weight, SES, parents’ education level	No significant association between increased screen time and risk of overweight or obesity.
J Nepal Health Res Counc/ 2018/ Saha M [31]	Bangladesh	School/ 288	Cross-sectional	10–14/ both	Obesity defined as ≥95th percentile of age	TV, Video game, PC	None	No significant association between increased screen time and risk of overweight or obesity.
Tropical Doctor/ 2018/ Mansouri N [42]	Pakistan	School/ 887	Cross-sectional	11–15/ both	Overweight defined as ≤95th and ≥ 85th percentile of age	TV	Age, sex, type of school, sleeping soft drink consumption	Watching TV more than 2 hours/ day was associated with increased risk of overweight (6.42 (4.32–9.54) *P* < 0.0001)
Prev Chronic Dis/ 2018/ Hu EY [74]	USA	School/ 15,624	Cross-sectional	14–18/ both	Obesity defined as ≥95th percentile of age	TV, Video or computer game, PC use	Age, sex, and race/ethnicity	Increased risk of obesity for those with more than 3 hours/ day TV watching (1.38 (1.09–1.76) and more than 3 hours video game or PC use (1.19 (0.98–1.43)
BMC Res Notes/ 2018/ Godakanda I [43]	USA	School/ 880	Cross-sectional	14–15/ both	Overweight defined as BMI Z-score ≥ 1	TV, Video/ DVD	Age, sex, ethnicity, schooling years	Television watching time ≥ 2 h/day (2.6 (1.7–3.8) and Video/DVD watching ≥2 h/day (3.1 (1.8–5.3) were associated with increased risk of overweight.
Egypt Ped Assoc Gazette/ 2016/ Talat MA [44]	Egypt	School/ 900	Cross-sectional	12–15/both	Overweight/ obesity defined as ≤95th and ≥ 85th and ≥ 95th percentile of age respectively	TV	Age, gender, SES	More than 2 hours TV watching was associated with increased risk of obesity (1.36 CI = 0.45–6.8; *P* = 0.048)
BMJ Open/ 2016/ Piryani S [75]	Nepal	School/ 360	Cross-sectional	16–19/ both	Overweight defined as BMI Z-score ≥ 1	TV	Age, sex, ethnicity, type of school, mother’s educational and occupation, family type, number of siblings, SES, watching TV and fruit consumption	Watching TV more than 2 hours/ day was associated with increased risk of obesity (OR = 8.86 (3.90 to 20.11) < 0.001
Med J Islamic Rep Iran/ 2016/ Moradi G [45]	Iran	School/ 2506	Cross-sectional	10–12/ both	Overweight/ obesity defined as ≤95th and ≥ 85th and ≥ 95th percentile of age respectively	TV, VG	Age, sex, SES	Screen time was associated with increased risk of overweight and obesity (1.41 (1.17–1.69)
Indian J Comm Health/ 2015/ Watharkar A [46]	India	School/ 806	Cross-sectional	12–15/ both	Overweight/ obesity defined as ≤95th and ≥ 85th and ≥ 95th percentile of age respectively	TV, PC, cell phone	None	Increased risk of overweight obesity for those with more than 2 hours TV watching (OR = 3.72; CI = 2.38–5.83) or more than 2 hours computer or mobile phone use (OR = 1.68; CI = 1.09–2.57)
Revista Paulista de Pediatria/ 2015/ De Lucena JMS [47]	Brazil	School/ 2874	Cross-sectional	14–19/ both	Overweight 25 ≤ BMI ≤ 30 kg/m^2^ and obesity BMI ≥ 30 kg/m^2^	TV, PC, VG	None	Excessive screen time was associated with increased risk of overweight/ obesity (1.25 (0.93–1.67)
BMC Pediatr/ 2014/ Velásquez-Rodríguez CM [48]	Finland	Community/ 120	Cross-sectional	10–18/ both	Overweight defined as ≤95th and ≥ 85th percentile of age	TV	None	Increased risk of overweight in excessive TV watchers among adolescents with insulin resistance (OR = 2.39; CI = 0.94–6.05) but not among healthy adolescents.
Int J Obes/ 2013/ De Jong E [40]	Netherland	School/ 2004 + 2068	Cross-sectional	10–13/ both	Overweight 25 ≤ BMI ≤ 30 kg/m^2^ and obesity BMI ≥ 30 kg/m^2^	TV, PC	Family characteristics and lifestyle nutrition behaviours	No significant association between TV watching more than 1.5 hours or PC use of more than 30 minutes and overweight/ obesity.
JCRPE/ 2012/ Ercan S [49]	Turkey	School/ 8848	Cross- sectional	11–18/ both	Overweight 25 ≤ BMI ≤ 30 kg/m^2^ and obesity BMI ≥ 30 kg/m^2^	TV, PC	None	Increased risk of overweight and obesity for those with more than 2 hours TV watching or PC use.
Pediatrics/ 2012/ Drake KM [50]	England	School/ 1718	Cross-sectional	12–18/ both	Overweight/ obesity defined as ≤95th and ≥ 85th and ≥ 95th percentile of age respectively	TV, DVD, video game	Adolescent demographics (gender, grade in school, race [white/nonwhite]);screen time; academic performance; employment status; diet quality (fast food, fruit and vegetable consumption over the past week)	Screen time of 7.1–14 and > 14 hours/week was associated with increased obesity risk of OR = 1.28 CI = 1.06, 1.55; *P* < 0.05 and OR = 1.37 CI = 1.09, 1.71; *P* < 0.01 respectively.
J Korean Med Sci/ 2012/ Byun W [53]	Korea	Community/ 1033	Cross-sectional	12–18/ both	Overweight/ obesity defined as ≥95th percentile of age	TV, PC, video game	Age, sex, annual household income, and moderate-to-vigorous physical activity	Increased risk of overweight and obesity was observed by increased screen time
Ital J Pediatr / 2012/ Adesina AF [51]	Nigeria	School/ 690	Cross-sectional	10–19/ both	Overweight/ obesity defined as ≤95th and ≥ 85th and ≥ 95th percentile of age respectively	TV	None	Increased risk of overweight and obesity was observed by increased screen time
Childhood Obesity/ 2011/ El-Gilany AH [52]	Egypt	School/ 953	Cross-sectional	14–19/ both	Overweight defined as ≤95th and ≥ 85th percentile of age	TV, PC	Age, sex, socioeconomic level, geographic area, ethnicity	Increased risk of overweight/ obesity for those with more than 2 hours TV watching (2.6 (1.7–3.9) or more than 2 hours computer use (1.8 (1.3–2.5)
J Epidemiol/ 2009/ Sun Y [32]	Japan	School/ 2842	Cross-sectional data of an original cohort	12–13/ both	Overweight 25 ≤ BMI ≤ 30 kg/m^2^	TV, VG	Age, parental overweight, and other lifestyle variables	Watching TV more than 3 hours/ d was associated with increased risk of overweight in boys (OR = 1.79; CI = 1.21–2.67 and girls OR = 2.37; CI = 1.55–3.62; *P* < 0.001
Int J Pediatr Obes/ 2008/ Collins AE [34]	Indonesia	School/ 1758	Cross-sectional	12–15/ both	Obesity defined as BMI ≥ 25 kg/m^2^	PC, PS	None	Increased risk of obesity in those with more than 3 hours/ d PC use (OR = 1.85; CI = 1.04–3.29) or play station use (OR = 1.94; CI = 1.23–3.05)

*Abbreviations*: *BMI* Body mass index, *TV* Television, *ST* Screen time, *SBP* Systolic blood pressure, *DBP* Diastolic blood pressure, *PC* Personal computer, *DVD* Digital video discs, *VCDs* Video compact disc digital, *SEP* Socioeconomic position, *SES* Socioeconomic status, *ST* Measurement in all of the studies was performed by questionnaire. All of the included participants were apparently healthy

### The results of the meta-analysis

In the current meta-analysis, after searching the electronic databases, a total of 6291 articles were retrieved (Fig. 1). After removing 2600 duplicated studies and 1535 records according to title/abstarct irrelevancy, 2156 artciles remained for final full-text screening. Then, 2112 manuscripts were removed due to not meeting the inclusion criteria. Finally, 44 manuscripts with a total number of 112,489 participants were included in the final meta-analysis. The included stdueis had a cross-sectional design and recruitted both genders.

**Fig. 2:**
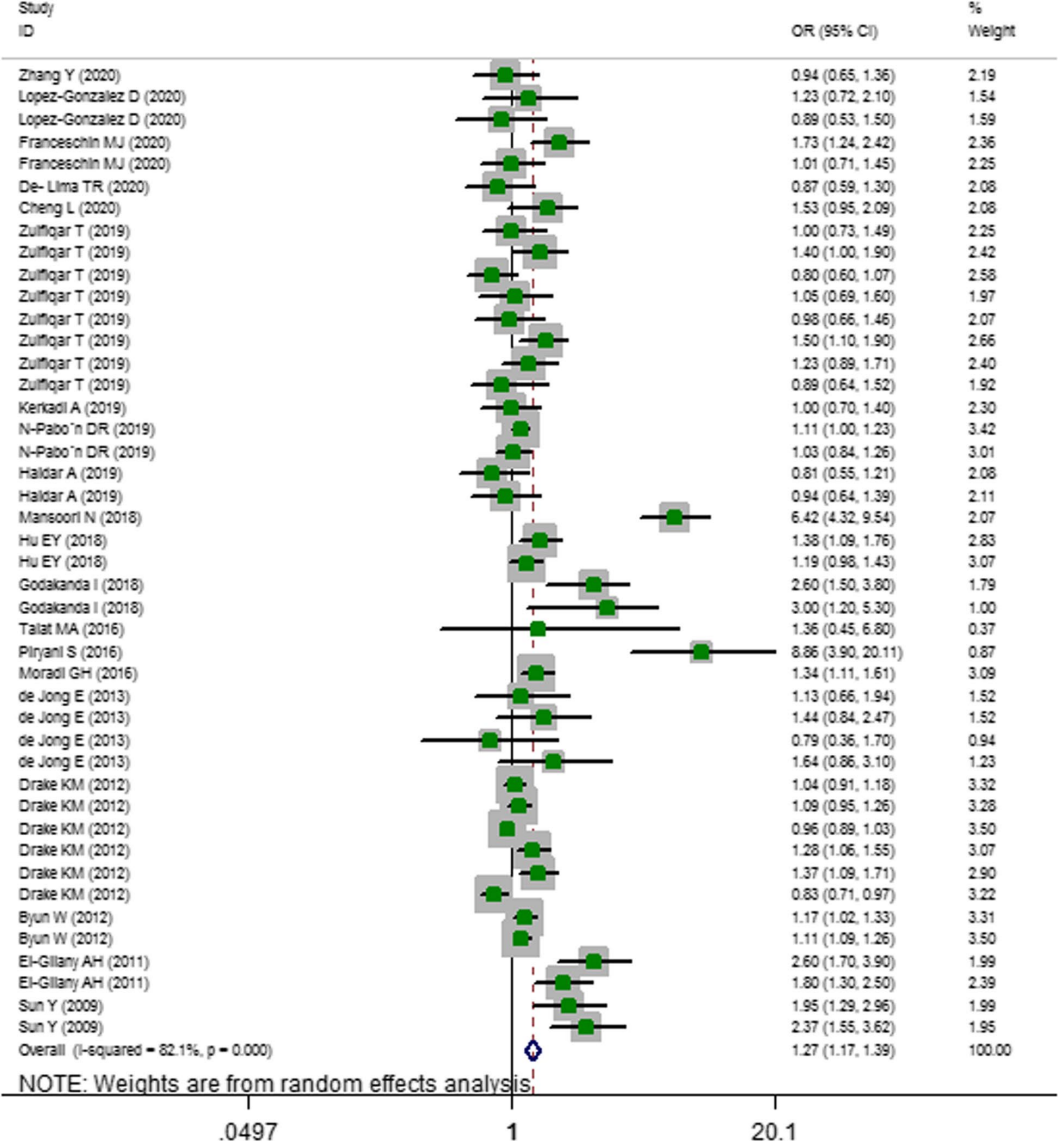
Odds ratio (OR) with 95% confidence interval (CI) of overweight/ obesity in highest versus lowest screen user adolescents. I^2^ represents the degree of heterogeneity

The results of the two-class meta-analysis is presented in Fig. 2. As can be seen, adolescents in the highest category of screen time were 1.27 times more likely to develop overweight/obesity compared to those in the lowest category (OR = 1.273; 95%CI = 1.166–1.390; *P* < 0.001; I-squared = 82.1%).

The results of subgrouping is shown in Table 4. Subgrouping according to continent reduced heterogeneity to some degree. For example, in the studies carried out in the United States, the heterogeneity reduced to 39.9%. Similarly, setting also was a possible source of heterogenity since subgropuing by setting reduced the heterogenity of community-based studies to 21.2%. However, other parameters were not potent sources of heterogeneity.

**Table 4:** Subgroup analysis for the odds of overweight/ obesity in highest versus lowest screen-user adolescents

Group	No. of studies^*^	OR (95% CI)	***P***_**within group**_	***P***_between group *_	***P***_**heterogeneity**_	I^**2**^, %
**Total**	44	1.273 1.166 1.390	< 0.001		< 0.001	82.1
**Continent**				< 0.001		
*America*	11	1.115 1.002 1.241	0.046		0.083	39.9
*Europe*	10	1.080 0.966 1.208	0.276		0.002	66.2
*Asia*	11	2.014 1.450 2.798	< 0.001		< 0.001	90.9
*Oceania*	8	1.099 0.927 1.304	0.278		0.056	49.1
*Africa*	4	1.646 1.018 2.660	0.042		< 0.001	86.9
**Screen type**				< 0.001		
*TV*	16	1.813 1.420 2.315	< 0.001		< 0.001	86.7
*PC*	3	1.467 0.950 2.265	0.509		0.159	45.7
*VG*	5	1.114 0.808 1.536	0.084		0.014	67.9
*TV + VG*	5	1.094 0.959 1.248	0.184		0.107	47.5
*VG + PC*	2	1.106 1.030 1.187	0.005		0.612	0
*TV + VG + PC*	13	1.068 0.974 1.172	0.163		0.002	60.7
**Age group**				< 0.001		
*< 15*	23	1.375 1.131 1.672	0.001		< 0.001	81.9
*≥ 15*	6	1.470 1.076 2.008	0.016		< 0.001	82.8
*Both*	15	1.126 1.032 1.228	0.008		< 0.001	76.4
**Setting**				< 0.001		
*School*	31	1.405 1.228 1.608	< 0.001		< 0.001	86.6
*Community*	13	1.109 1.040 1.182	0.002		0.229	21.2
**Obesity status**				< 0.001		
*Obesity*	11	1.109 0.964 1.275	0.150		0.001	67.0
*Overweight*	9	1.567 1.282 1.916	< 0.001		< 0.001	84.1
*Overweight/ obesity*	24	1.271 1.105 1.463	0.001		< 0.001	87
***Sample size***				< 0.001		
*1000 >*	11	2.024 1.303 3.144	0.002		< 0.001	90.9
*1000–5000*	27	1.121 1.049 1.198	0.001		< 0.001	59.9
*≥ 5000*	6	1.323 1.017 1.722	0.037		0.001	75.7
Study quality *				< 0.001		
*Low*	0	–		–	–	–
*Moderate*	31	1.259 1.085 1.461	< 0.001		< 0.001	80.3
*High*	13	1.282 1.146 1.435	0.002		< 0.001	84.4
**Adjusted covariates**				< 0.001		
*Age, sex, nationality, SES*	7	1.239 1.116 1.377	< 0.001		0.586	0
*Age, sex, nationality, SES, other demographic variables*	14	1.454 1.251 1.690	< 0.001		< 0.001	87.3
*Age, sex, nationality, SES, other demographic variables, dietary habits*	23	1.091 0.982 1.212	0.107		< 0.001	64.8

*low quality = 0–3; moderate quality = 4–7; high quality ≥8; all of the included studies were in moderate quality group therefore, subgrouping was not performed

**Fig. 3:**
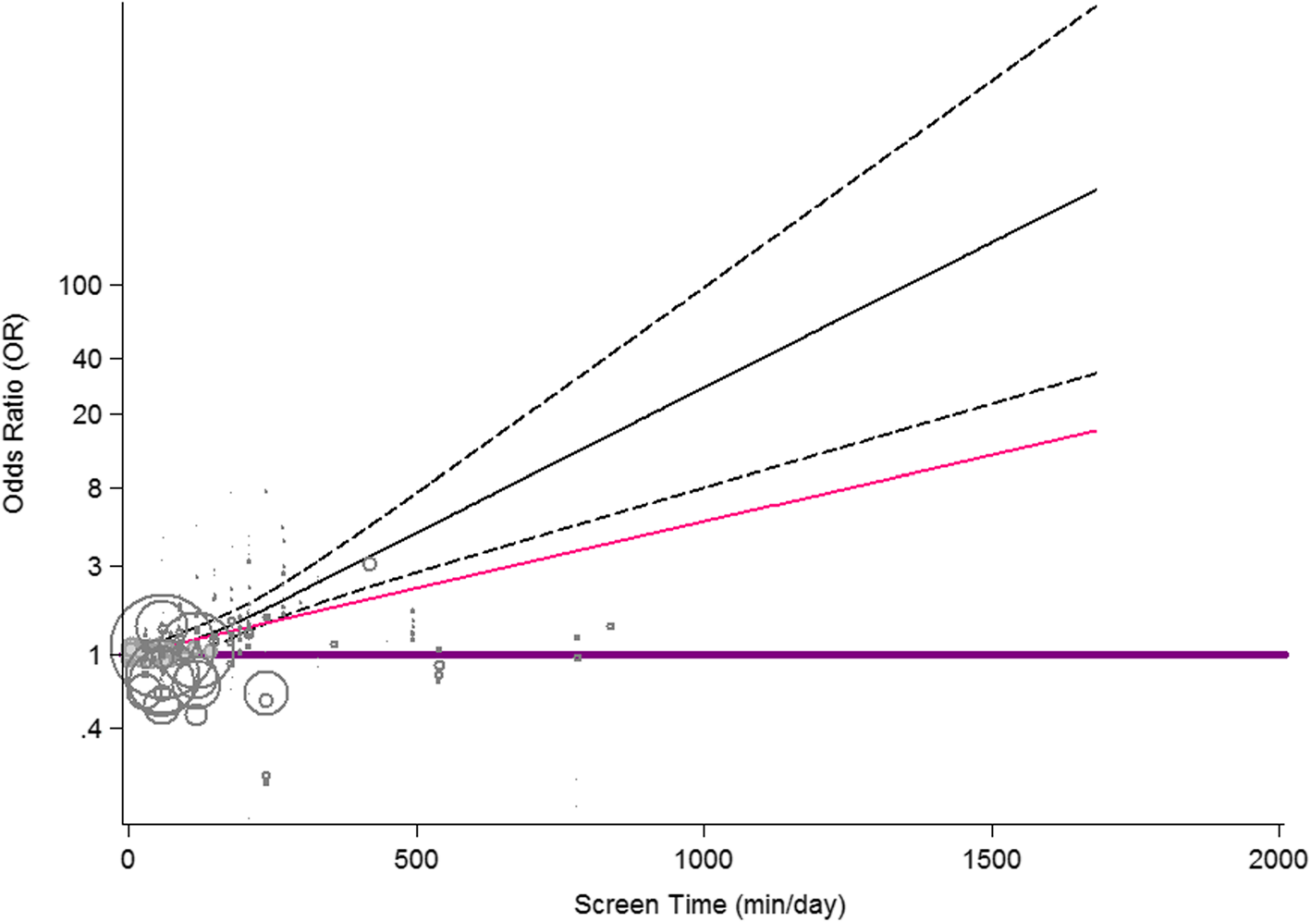
Dose–response association between screen time and odds of overweight/ obesity. Linear relation (solid line) and 95% CI (dashed lines) of pooled OR of obesity by 1 *min*/*day* increment of screen time (p- nonlinearity = 0.310) among adolescents

The results of dose-response relationship between screen time and overweight/obesity is presented in Fig. 3. There was no evidence of non-linear association between increased screen time and risk of overweight/obesity (P-nonlinearity = 0.311).

**Fig. 4:**
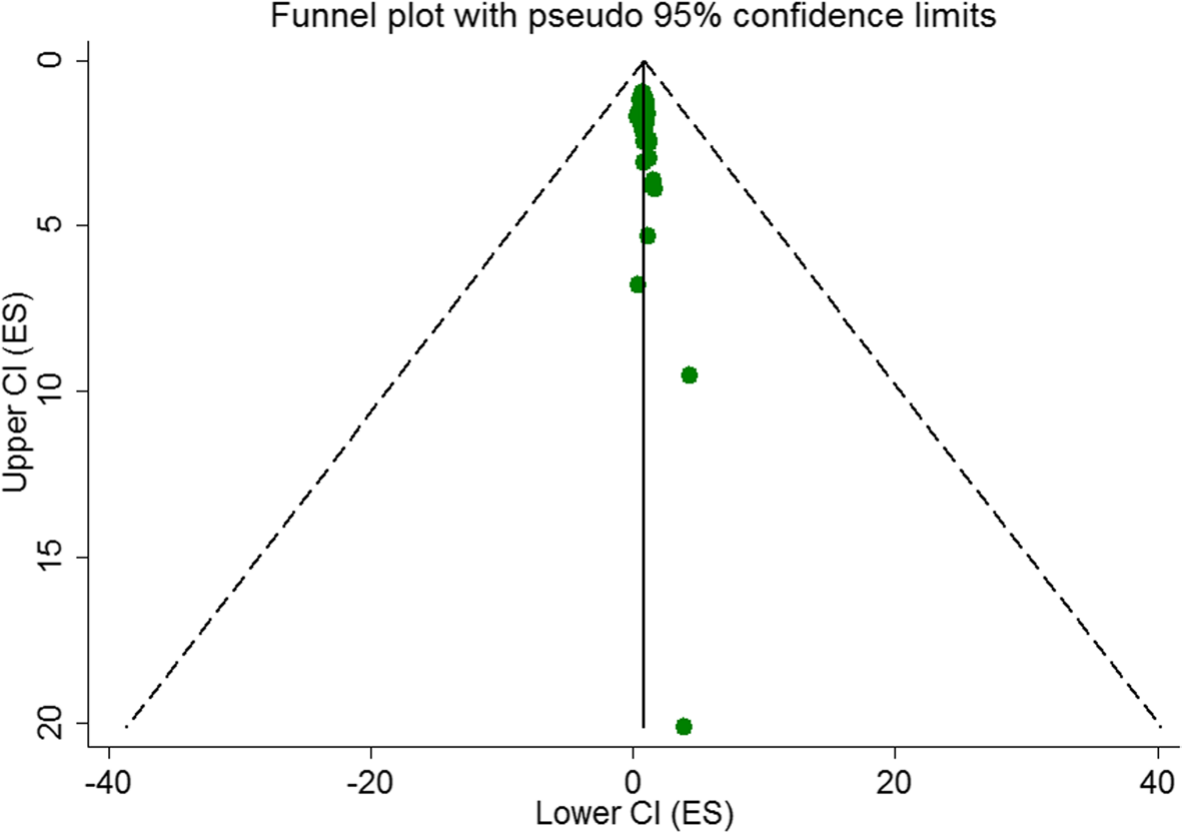
Begg’s funnel plot (with pseudo 95% CIs) of the odds of overweight/ obesity in highest versus lowest screen time categories among adolescents

**Fig. 5:**
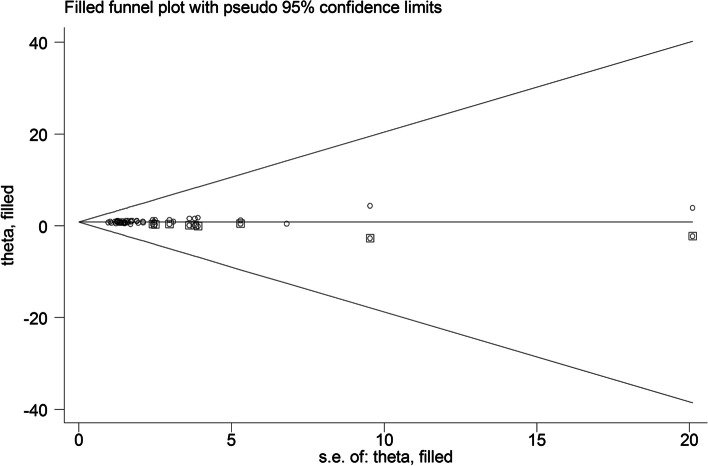
Filled funnel plot with pseudo 95% confidence limits for studies evaluating the association between screen time and overweight/ obesity among adolescents [OR = 1.472; CI = 1.083, 2.068; *P* < 0.001]

Funnel plots indicating publication bias are presented in Fig. 4. The results of Begg’s and Egger’s tests showed some evidence of publication bias (Egger’s *P*-value = 0.001; Begg’s *P*-value = 0.001). Therefore, trim-and-fill analysis was performed (Fig. 5) and the obtained results were reported (95%OR = 1.472; 95% CI = 1.083–2.068; *P* < 0.001).

## Discussion

In the current meta-analysis, for the first time, we summarized the results of studies that evaluated the association between screen time and overweight/obesity risk among adolescents. In addition, in a large sample size (*n* = 112,489), we witnessed that high screen time was associated with 1.27-time higher chance of overweight/obesity among adolescents. No evidence of non-linear association was observed in the dose-response analysis.

Previous population-based studies have revealed the obesity-promoting effects of high screen time. Lopez-Gonzalez [28] evaluated more than 7511 registered schools and reported that high screen time was considered as an obesogenic factor. Several other studies also revealed that screen time more than two or 3h per day increased the risk of obesity [26, 33]. Internet addicted adolescents had also elevated risk of obesity in one study [27]. However, several other studies reported no significant association between obesity and screen time [30, 35, 41]. The possible strong reason for this inconsistency might be attributed to the type of screen (e.g., TV, video games, PCs, etc.) used in different studies.

In this study, we also performed subgroup analysis. According to the results, the studies that defined video games as their screen failed to reveal a positive association between screen time and obesity [31, 39, 41]. In our meta-analysis, video games alone or in combination with other screen types failed to show a positive obesity-promoting effect. In the study by Sun et al. [32], the positive association between video game playing and risk of obesity was only observed among girls and not boys. Zulfiqar et al. [33], also reported the positive association between obesity and TV watching, but not for video games. Several studies even showed the negative association between active video games and obesity. In the study by Strahan et al. [76], active video games reduced the chance of obesity among adolescents. This is possibly because some video games can increase physical activity and physical health. In a meta-analysis by Primack et al., video games were associated with 69% improve in psychological therapy outcomes and 50% improve in physical activity outcomes [77]. In another study by Williams, active video games were introduced as effective tools to improve physical activity among adolescents and were considered as a more acceptable and sustainable approach than many conventional methods [78].

In our meta-analysis, the most important obesogenic screen was TV (OR = 1.813; 95%CI: 1.420–2.315, *P* < 0.001). In the study by Franceschin et al. [22], adolescents watching TV for more than 2h per day had almost doubled chance of being obese compared to those watching TV for less than 2h per day (OR = 1.73; 95% CI = 1.24–2.42); but the association was not significant for playing video games or using the PC. Therefore, it seems that TV watching is a stronger motivator of obesity among adolescents. Also, the age group is a determinant of screen type use and the consequent obesity. In our study, most of the included studies had been performed in adolescents less than 15 years old and the association between screen time and overweight/obesity in this age group was stronger (*P* < 0.001) because at lower ages, children and adolescents have less structured time than older adolescents and most of this unstructured time is filled by watching TV [51, 79]. Another important finding in our subgrouping was the role of setting. In school-based studies, the association between screen time and overweight/obesity was more pronounced than other study types (OR = 1.405; 95% CI: 1.228–1.608; *P* < 0.001) because adolescents usually have more tendency to eat calorie foods in restaurants. Most of the adolescents buy lunch at school canteen and restaurants and are more likely to develop overweight and obesity [80].

High screen time, as a sedentary behavior, reduces lipoprotein lipase activity (LPL) and leads to reduced plasma triglycerides’ absorption by skeletal muscles, reduced HDL level and postprandial increase in serum lipids, that consequently results in fat deposition in vessels or adipose tissue [81–83]. Moreover, increased screen time increases food intake. Previous studies revealed that television watching increases motivated response to food intake and snacking behavior among children and adolescents [4, 84–87]; this is also true for video games [88–90] and personal computer use [91, 92]. More importantly, several TV food advertisements promote the consumption of junk food and fast foods and increase the risk of obesity [93–98]. Therefore, the association between obesity and screen use is a multi-dimensional factor. Also, the results of included studies in our meta-analysis were reported for both genders; therefore, it was not possible to give gender-specific results.

This study had some limitations. First, this study had a cross-sectional design, which precludes causal inference. Second, the data collection method for screen time measurement was self-reported questionnaires that might be biased. Third, we were not able to perform subgroup analysis for some important confounders, such as eating food while watching screen, type of video games (active or non-active), and gender because the articles had not mentioned such information.

However, this is the first meta-analysis reporting the association between screen time and overweight/obesity among adolescents. We raised concerns among parents, health care professionals, educators, and researchers about the effects of screen time on the health of adolescents. Our study has some important clinical and health implications for policy makers to develop strategies to encourage adolescents to be more physically active and to apply some restrictions for school-based meal servings. They can also improve access of adolescents to opportunities for physical activity, as is the case with state laws related to the quantity and quality of physical education. Also, parents should pay more attention to the adolescents’ screen-based behaviors and apply some at-home restrictions. Setting restrictions on screen use at certain times is a great way to protect adolescents from potentially harmful online activities and encourages them to use their time appropriately.

## Conclusion

The current meta-analysis is the first study providing quantitative results for the association between different screen types and overweight/obesity among adolescents. Further studies are warranted to focus on the effects of gender, different screen types and video games to better explain the discrepancies in the obtained results.

## Supplementary Information

**Additional file 1.**

## Data Availability

The data that support the findings of this study are available from Tabriz University of Medical Sciences but restrictions apply to the availability of these data, which were used under license for the current study, and so are not publicly available. Data are however available from the authors upon reasonable request and with permission of corresponding author.
