# Supplementary material for: Screen time increases overweight and obesity risk among adolescents: a systematic review and dose-response meta-analysis
Source: BMC Prim Care. 2022 Jun 28;23:161. doi: 10.1186/s12875-022-01761-4 (PMC9238177; doi:10.1186/s12875-022-01761-4)
Supplement: Supplementary file 1 — Additional file 1. [file 12875_2022_1761_MOESM1_ESM.docx]

**Title: Screen time increases overweight and obesity risk among adolescents: A systematic review and dose-response meta-analysis**

Purya Haghjoo ^1^, Goli Siri ^2^, Ensiye Soleimani ^3^, Mahdieh Abbasalizad Farhangi ^4^, Samira Alesaeidi ^5^

^1^ Urology Research Center, Razi Hospital, School of Medicine, Guilan University of Medical Sciences, Rasht, Iran.

^2^ Department of Internal Medicine, Amir Alam Hospital, Tehran University of Medical Sciences, Tehran, Iran.

^3^ Student Research Committee, Tabriz University of Medical Sciences, Tabriz, Iran.

^4^ Department of Community Nutrition, Faculty of Nutrition, Tabriz University of Medical Sciences, Tabriz, Iran.

^5^ Rheumatology Research Center, Tehran University of Medical Sciences, Tehran, Iran.

***Corresponding Author**, Email address: [abbasalizadm@tbzmed.ac.ir](mailto:abbasalizadm@tbzmed.ac.ir), [abbasalizad_m@yahoo.com](mailto:abbasalizad_m@yahoo.com), Tel: +98 0413 3357580

**Registration:** The protocol of the current work has been registered in the PROSPERO system (Registration number: CRD42021233899).

**Supplementary Material**

**Sup. Table 1. PRISMA Checklist [1]**

| **Section/topic** | **#** | | | **Checklist item** | **Reported on page #** |
| --- | --- | --- | --- | --- | --- |
| **TITLE** | | | | |  |
| Title | 1 | | | Identify the report as a systematic review, meta-analysis, or both. | Page 1; lines 2-3 |
| **ABSTRACT** | | | | |  |
| Structured summary | 2 | | | Provide a structured summary including, as applicable: background; objectives; data sources; study eligibility criteria, participants, and interventions; study appraisal and synthesis methods; results; limitations; conclusions and implications of key findings; systematic review registration number. | Page 2; lines 2-15 |
| **INTRODUCTION** | | | | |  |
| Rationale | 3 | | | Describe the rationale for the review in the context of what is already known. | Pages; lines 6-19 |
| Objectives | 4 | | | Provide an explicit statement of questions being addressed with reference to participants, interventions, comparisons, outcomes, and study design (PICOS). | Page 3; lines 15-19 |
| **METHODS** | | | | |  |
| Protocol and registration | 5 | Indicate if a review protocol exists, if and where it can be accessed (e.g., Web address), and, if available, provide registration information including registration number. | | | Page 4; lines 21-23 |
| Eligibility criteria | 6 | Specify study characteristics (e.g., PICOS, length of follow-up) and report characteristics (e.g., years considered, language, publication status) used as criteria for eligibility, giving rationale. | | | Page 5; lines 3-10 |
| Information sources | 7 | Describe all information sources (e.g., databases with dates of coverage, contact with study authors to identify additional studies) in the search and date last searched. | | | Page 4; lines 25-26 |
| Search | 8 | Present full electronic search strategy for at least one database, including any limits used, such that it could be repeated. | | | Page 5; lines 1-2 and Sup.Table 2 |
| Study selection | 9 | State the process for selecting studies (i.e., screening, eligibility, included in systematic review, and, if applicable, included in the meta-analysis). | | | Page 4; lines 25-26  Page 5; lines 1-10 |
| Data collection process | 10 | Describe method of data extraction from reports (e.g., piloted forms, independently, in duplicate) and any processes for obtaining and confirming data from investigators. | | | Page 5; lines 11-18 |
| Data items | 11 | List and define all variables for which data were sought (e.g., PICOS, funding sources) and any assumptions and simplifications made. | | | Page 5; lines 19-25  Page 6; lines 1-6 |
| Risk of bias in individual studies | 12 | Describe methods used for assessing risk of bias of individual studies (including specification of whether this was done at the study or outcome level), and how this information is to be used in any data synthesis. | | | Page 5; lines 11-18 |
| Summary measures | 13 | State the principal summary measures (e.g., risk ratio, difference in means). | | | Page 6; lines 5-26  Page 6; lines 1-3 |
| Synthesis of results | 14 | Describe the methods of handling data and combining results of studies, if done, including measures of consistency (e.g., I^2^) for each meta-analysis. | | | Page 6; lines 5-26  Page 6; lines 1-3 |
| Risk of bias across studies | 15 | | Specify any assessment of risk of bias that may affect the cumulative evidence (e.g., publication bias, selective reporting within studies). | | Table 4  Page 6; lines 16-20 |
| Additional analyses | 16 | | Describe methods of additional analyses (e.g., sensitivity or subgroup analyses, meta-regression), if done, indicating which were pre-specified. | | Table 4  Page 6; lines 16-20 |
| **RESULTS** | | | | |  |
| Study selection | 17 | | Give numbers of studies screened, assessed for eligibility, and included in the review, with reasons for exclusions at each stage, ideally with a flow diagram. | | Page 7; lines 6-23  Table 3 |
| Study characteristics | 18 | | For each study, present characteristics for which data were extracted (e.g., study size, PICOS, follow-up period) and provide the citations. | | Table 3 |
| Risk of bias within studies | 19 | | Present data on risk of bias of each study and, if available, any outcome level assessment (see item 12). | | Table 2 |
| Results of individual studies | 20 | | For all outcomes considered (benefits or harms), present, for each study: (a) simple summary data for each intervention group (b) effect estimates and confidence intervals, ideally with a forest plot. | | Figure 2, 3, 4  Page 7; lines 25-26  Page 8; lines 1-19 |
| Synthesis of results | 21 | | Present results of each meta-analysis done, including confidence intervals and measures of consistency. | | Figure 2, 3, 4 |
| Risk of bias across studies | 22 | | Present results of any assessment of risk of bias across studies (see Item 15). | | Figure 4, 5 |
| Additional analysis | 23 | | Give results of additional analyses, if done (e.g., sensitivity or subgroup analyses, meta-regression [see Item 16]). | | Figure 4, 5 |
| **DISCUSSION** | | | | |  |
| Summary of evidence | 24 | | Summarize the main findings including the strength of evidence for each main outcome; consider their relevance to key groups (e.g., healthcare providers, users, and policy makers). | | Pages 8; lines 20-25 |
| Limitations | 25 | | Discuss limitations at study and outcome level (e.g., risk of bias), and at review-level (e.g., incomplete retrieval of identified research, reporting bias). | | Page 10; lines 20-24 |
| Conclusions | 26 | | Provide a general interpretation of the results in the context of other evidence, and implications for future research. | | Page 11; lines 11-15 |
| **FUNDING** | | | | |  |
| Funding | 27 | | Describe sources of funding for the systematic review and other support (e.g., supply of data); role of funders for the systematic review. | | Page 11; lines 16, 17 |

| **Search strategy** | **Database** | **Num. of records** |
| --- | --- | --- |
| ((((((((((((obesity[MeSH Terms]) OR (central obesity[MeSH Terms])) OR (overweight[MeSH Terms])) OR (body mass index[MeSH Terms])) OR (abdominal obesity[MeSH Terms])) OR (overweight[MeSH Terms])) OR (waist circumference[MeSH Terms])) OR (ratio, waist to hip[MeSH Terms])) OR (body fat distribution[MeSH Terms])) OR (abdominal fat[MeSH Terms])) OR(body composition [MeSH Terms])) AND ((((((((((((sedentary behav*[MeSH Terms]) OR (screen time[MeSH Terms])) OR (sitting time[MeSH Terms])) OR (sitting time[MeSH Terms])) OR (television view*[MeSH Terms])) OR (watching television[MeSH Terms])) OR (computer use[MeSH Terms])) OR (internet use[MeSH Terms])) OR (smart phone[MeSH Terms])) OR (video game*[MeSH Terms])) OR (electronic game*[MeSH Terms])) OR (depress*[MeSH Terms]))) AND (((((((((((Child*[MeSH Terms]) OR (children[MeSH Terms])) OR (teen*[MeSH Terms])) OR (adolescent[MeSH Terms])) OR (boy*[MeSH Terms])) OR (girl[MeSH Terms])) OR (all, childhood[MeSH Terms])) OR (pediatric*[MeSH Terms])) OR (youth[MeSH Terms])) OR (teenager[MeSH Terms])) OR (toddler*[MeSH Terms])) | PubMed | 2121 |
|  | Scopus | 2016 |
|  | Embase | 2154 |

**Sup. Table 2**. Search strategies and the number of records according to different electronic database

**References**

1. Moher, D., et al., *Preferred reporting items for systematic reviews and meta-analyses: the PRISMA statement.* Annals of internal medicine, 2009. **151**(4): p. 264-269.
